# Cryptic Genetic Variation in Evolutionary Developmental Genetics

**DOI:** 10.3390/biology5020028

**Published:** 2016-06-13

**Authors:** Annalise B. Paaby, Greg Gibson

**Affiliations:** School of Biology, Georgia Institute of Technology, Atlanta, GA 30332, USA; annalise.paaby@biology.gatech.edu

**Keywords:** evolution, development, genetics, quantitative genetics, cryptic genetic variation

## Abstract

Evolutionary developmental genetics has traditionally been conducted by two groups: Molecular evolutionists who emphasize divergence between species or higher taxa, and quantitative geneticists who study variation within species. Neither approach really comes to grips with the complexities of evolutionary transitions, particularly in light of the realization from genome-wide association studies that most complex traits fit an infinitesimal architecture, being influenced by thousands of loci. This paper discusses robustness, plasticity and lability, phenomena that we argue potentiate major evolutionary changes and provide a bridge between the conceptual treatments of macro- and micro-evolution. We offer cryptic genetic variation and conditional neutrality as mechanisms by which standing genetic variation can lead to developmental system drift and, sheltered within canalized processes, may facilitate developmental transitions and the evolution of novelty. Synthesis of the two dominant perspectives will require recognition that adaptation, divergence, drift and stability all depend on similar underlying quantitative genetic processes—processes that cannot be fully observed in continuously varying visible traits.

## 1. Introduction

This paper offers a quantitative genetics perspective on evolution and development. The thoughts have been shaped by our post-doctoral experiences two decades apart, working with *Drosophila melanogaster* and *Caenorhabditis elegans*, attempting to bridge the gap between micro- and macro-evolution [1,2]. We start with two sets of observations, namely that developmental systems are simultaneously robust and labile, and that standard statistical genetic models at the species level are not really sufficient to explain major evolutionary transitions. Next, the bulk of the paper argues that recent genetic analyses of the architecture of complex traits need to be integrated with developmental perspectives, and that, when that is done, the importance of cryptic genetic variation becomes apparent. We conclude with some thoughts both on the capacity of organisms to rapidly adapt to environmental change, as well as the implications of developmental system drift for the origin of evolutionary novelties.

**Figure 1:**
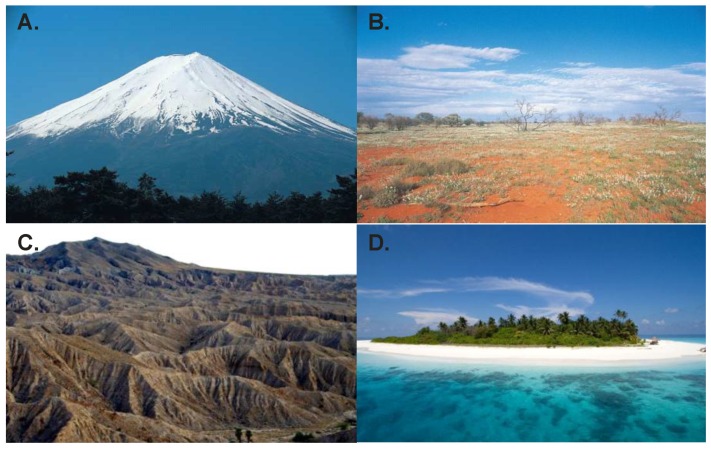
Four metaphors for the adaptive landscape. (**A**) Fisherian single adaptive peak; (**B**) Kimuran nearly flat landscape; (**C**) Wrightian rugged landscape; (**D**) an island peak where what is below the surface matters.

In order to place all of this in the broader perspective of evolutionary genetics, we present Figure 1 as a series of metaphors for different views of adaptation. Figure 1A is the standard model due to Fisher [3] of an adaptive landscape dominated by a single fitness peak which needs to be climbed by gradual fixation, predominantly of newly arising mutations that confer a natural selective advantage, as modeled influentially by Orr [4]. Figure 1B is the neutralist perspective, initially enunciated by Kimura [5] to explain patterns of evolution of protein sequences, which does not deny the existence of fitness peaks, but does emphasize that a considerable amount of evolution at the DNA sequence level is at least nearly neutral. Figure 1C argues, in the tradition of Wright [6], for a more nuanced perspective that adaptive landscapes are somewhere between hilly and rugged: Organisms already occupy peaks, and whether or not they can traverse to even higher ground is a function of the depth of valleys in between and the willingness of explorers to attempt the crossing. Without suggesting that our worldview is anywhere near as developed as these notions, Figure 1D simply presents a fourth metaphor, which is meant to emphasize that the landscape we see above the water is not necessarily what will shape the long-term future of a species. We considered an iceberg, since the majority of their substance is hidden, but since they float in their environment and what is underneath may not matter, we instead present an island in the Maldives, whose fate in the face of rising sea-levels has everything to do with what is not yet seen. Hidden, or cryptic, genetic variation in our view is the key to long-term survival and evolutionary transition.

### 1.1. Robustness, Plasticity and Lability

One of the more remarkable features of developmental systems is their robustness to perturbation [7]. At the species level, this is seen as the general tendency for organisms to develop normally despite the insults, environmental or genetic, that may arise during ontogeny. Somehow genetic systems are able to produce healthy individuals with symmetric, well-coordinated organ systems even across wide ranges of temperature, nutrition, or when new genetic material is introduced by admixture or mutation [8]. Runting in domestic animals, minute flies that are half the normal size, and miniaturization of many plants demonstrate the ability of developmental systems to adjust to circumstances. Above the species level, robustness is seen in the persistence of developmental genetic mechanisms across taxa [9]. Wherever we look, conserved molecular pathways underpin the development of homologous traits: vertebrate body patterning, organogenesis, and neuronal pathfinding all trace back hundreds of millions of years, in many cases also being clearly identifiable in invertebrates [10]. Kaufman [11] has argued that this robustness is often embedded in the logic of regulatory pathways that, once evolved, entrench development, although ongoing stabilizing selection may help to ensure robustness.

A corollary to robustness is plasticity, namely the capacity to develop different, but predictable, morphs under different growth conditions. Whether it is social insect castes, fish adopting different morphs and behaviors, or plants following a so-called norm of reaction along a cline of geographic variation, species have the ability to modulate what is normal. Often times, two or more morphs are themselves robust, indicating that stable developmental systems can exist in the same species side-by-side, posing a considerable challenge for genetic modeling of complex trait evolution [12]. Ecologists recognize generalist and specialist species, and model the evolution of strategies that allow development (and behavior) to follow either more static or more plastic trajectories.

Although developmental systems are robust, they also demonstrate lability, the capacity to evolve rapidly. Adaptive radiations, for example of the Lake Malawi cichlids [13], illustrate how very different morphologies can quickly appear in a matter of thousands of generations. More generally, lability refers to the observations that despite strong conservation of developmental systems, the detailed interactions among component parts can and do evolve [14]. Mechanisms of sex determination are remarkably labile, involving changes at both the chromosomal level and in the key sex determination loci. Embryonic patterning is generally recognized to rely on highly conserved phylotypic states [15] that are nearly invariant for Classes of species, but can be arrived at through very different mechanisms: for example, determinant and indeterminant cell lineages in nematodes [16] and short- and long-germband patterning in insects [17]. Furthermore, as developmental geneticists dissect the regulatory regions of key patterning genes, notably the *fushi tarazu* pair-rule segmentation gene in *Drosophila*, they see that there is lability in the precise array of transcription factor binding sites across species [18].

### 1.2. Incompleteness of Standard Models

These twin properties of robustness and lability pose challenges for the standard genetic model of evolution, which posits that stabilizing selection maintains limited variability in populations, and that this is punctuated by periods of directional selection when a species explores a novel ecological niche. Micro-evolution concerns itself with the quantitative and population genetic processes that shape variation within a species, and it is generally assumed that macro-evolutionary trends are due to extrapolation of the same processes across time, supplemented by mechanisms that promote speciation [19]. The role of mutations of large effect in divergence continues to be debated, and while few authors suppose that hopeful monsters in the sense of Goldschmidt [20] are generally important, the focus of the literature on large effect genes underlying QTL, as well as the theory of adaptive walks, suggests that many believe that de novo mutations (rather than standing allelic variants) are critical for major evolutionary transitions to occur. However, the infinitesimal model now seems to fit the genetics of the vast majority of quantitative traits, with thousands of loci needed to explain even half the genetic variance of morphological, physiological, and behavioral traits alike [21].

We certainly do not mean to imply that micro-evolutionary processes are irrelevant to macro-evolution. Rather, we think that the importance of standing variation is if anything under-appreciated [22]. On the one hand, it provides a vast pool of variation that will facilitate rapid response to changed environmental or genetic circumstances, and on the other, much of it is of questionable relevance to adaptation since it is “conditionally neutral”, and protected by the very complexity of genetics that genome-wide association strategies have emphasized [23]. We are also mindful of the consideration that many evolutionary transitions are not so obviously explained by directional selection: Halteres are exquisite flight balancing organs that are onto-and phylo-genetically related to hindwings, but we do not see intermediates; and it is very easy to imagine how human morphology evolved from primate ancestors, but not so clear how higher intelligence was shaped by selection. Evolutionary novelties such as these are the result of the evolution of robust and labile developmental systems, perhaps assisted by plasticity and modularity. We need a much more sophisticated understanding of developmental quantitative genetics than that offered by hard selection on visible, major-effect mutations if we are to arrive at a complete picture of macroevolution.

## 2. The Quantitative Genetics of Developmental Systems

### 2.1. The Infinitesimal Model Dominates

How does natural genetic variation produce differences among individuals, and how does it facilitate the evolution of populations? This is the business of the fields of quantitative genetics and population genetics, both of which predate modern experimental methods in sequencing and molecular genetics by decades. Important early work in these fields established empirical and theoretical foundations to evolutionary biology under the assumption that natural genetic variation was comprised of near-infinite numbers of micro-mutations. In his challenge to evolutionary biologists, Rockman [24] points out that as contemporary investigations pursuing the genetic basis of adaptation have optimistically chased after large-effect alleles, this view of the infinitesimal model has been abandoned. However, this abandonment, driven by discovery bias, may not be justified.

When adaptive alleles have been identified in natural populations, they are more likely to have been discovered in candidate-gene approaches than in unbiased surveys of the genome [24,25]. Most often, however, attempts to identify genes (never mind causal mutations) that underlie variation and adaptation in wild populations fail. Thus, the list of adaptation alleles now known for *Drosophila*, *C. elegans*, mice, *Arabidopsis*, *Mimulus*, sticklebacks, yeast, bacteria, humans, and many other systems [26] may not be representative of the true stuff of evolution [24]. Even as the list incrementally grows, our bias to publish “findings” betrays the productivity of the effort. Adaptations underlying domestication provide some exceptions to this rule, presumably because exceptionally strong selection pressures have pushed fixation of major-effect alleles that would otherwise be deleterious. Evolution of maize from teosinte has been characterized by a handful of genes [27]; within species, independent mutations in *myostatin* underlie selection for muscle mass in cattle [28], just as insects have evolved resistance to insecticides in the same target gene [29].

The pervasiveness of polymorphisms of small effect is clearly illustrated in another maize experiment, the famous long-term response to selection. Initiated in 1896, the experiment selects on oil content, protein content and other traits and has yielded new strains with extreme values compared to the progenitors; it continues today and shows little sign of slowing [30]. Only a myriad of loci, each contributing small effects, can explain this progression. Years ago, Lewontin proposed the problem of the fecund female: Absurdly fecund flies that would theoretically produce two billion offspring if you could combine all of the projected polymorphism thought to underlie observed fecundity into a single female [31]. Is such a thing possible? Maybe, if the improbability of generating thousands of homozygotes for the right alleles in a single animal could be overcome. Contemporary analyses of highly quantitative traits illustrate similar phenomena. For example, genome-wide association studies link phenotype to narrow regions of the genome and show that these hits explain tiny fractions of the observed variation [32]. We now know that well over 1000 loci each contribute on the order of one millimeter to human height, and probably 10,000 loci a fraction of a millimeter [33]. If they could all be brought together, in theory we would see people several meters tall. Such a range of height is seen in dogs, where Chihuahuas and Great Danes stand at opposite ends of the spectrum. These differences are mostly attributed to a handful of very large effect growth-regulating alleles [34], but the infinitesimal background provides a vast pool of variation available to modify the phenotype, offset deleterious effects, and facilitate the adaptive walk. The studies of human height, like analyses of genomic prediction in agriculture, genotypic risk scoring in human disease, and estimates of genomic heritability, explicitly assume the infinitesimal model by considering contributions from every site [35,36,37,38]. The recent success of these high-investment research programs further confirms that the genetic basis of complex traits is highly polygenic and comprised of near-infinitesimal-effect loci.

### 2.2. Selection is Generally from Standing Variation

Newly arising quantitative trait alleles with tiny effect sizes have very little potential to contribute to adaptive evolution. This is because the birth of a new mutation occurs at the lowest possible frequency in a population, and with only a weakly beneficial effect an allele is more likely to be lost by chance than to become established. However, even as much of the theory explored by population genetic models relies on the premise that adaptation proceeds from new mutations, much of the empirical data from natural populations demonstrate evolutionary responses to standing genetic variation. For example, several decades of research in *Drosophila* has produced a staggering literature on clinal variation, in which populations show local adaptation via shifts in allele frequency along environmental gradients [39]. In humans, shifts in allele frequencies are often “soft”, implicating high polygenicity and small effect sizes in the underlying trait architecture [40,41]. Standing variation offers better potential for rapid evolutionary responses, and recent work has characterized cyclical changes in standing allele frequency in the face of seasonal selection pressures [42,43]. However, the role of new mutations is not to be trivialized; they are, after all, the source of all variation, and populations exhibiting clinal variation in allele frequency show a preponderance of derived (as opposed to ancestral) alleles associated with newly colonized habitats [44]. As such, the continuum between “new” and “standing” is defined by when, why and how: When after their arrival in a population do beneficial alleles contribute to adaptation, why do they accumulate, and how are they recognized by selection. As we describe here, the exposure of new or existing alleles to different environments, or their recombination onto different genetic backgrounds, plays a central role in their penetrance to phenotype.

### 2.3. Pleiotropy is Ubiquitous

If the infinitesimal model predominates, and most traits are highly polygenic, arising from the minute contributions of many loci, then by logical extension pleiotropy is extremely pervasive. (Fisher’s infinitesimal model [3] was a mathematical abstraction, and in fact assumed no pleiotropy, but rather an infinite number of contributing alleles; but the finite number of loci in real genomes, compared to the myriad number of phenotypes that may be defined by experimentalists, forces the conclusion that many genic elements affect many aspects of biological systems.) However, if allelic effects on phenotype are nearly infinitesimal, obviously our ability to detect them is limited. Investigations into the extent of pleiotropy are doubly plagued by limitations, because a phenotype unmeasured is a phenotype uncounted and because the requirement to detect multiple significant effects produces a systematic bias against observing pleiotropy. However, current disagreement over whether pleiotropy is pervasive [45,46] or restricted [47,48] is really about whether small effects are biologically significant [45]; in part a question of statistics, but also a question of the veracity of the infinitesimal model [49].

Identifying genic elements with pleiotropic effects on phenotype may be a challenge in the laboratory, but the reach of natural selection is far longer. From an evolutionary perspective, whether a single gene has multiple functions (molecular gene pleiotropy) or a mutant allele affects two or more traits (developmental pleiotropy) is not strictly relevant. All that matters is whether a mutation affects more than one component of fitness (selectional pleiotropy), since this is what natural selection sees [46]. For example, a genetic variant segregating in a population may increase male fitness, by acting through spermatogenesis, for example, but decrease female fitness, by acting through female-specific tissues; the fate of the allele will depend upon its relative contributions to fitness via these two separate traits, along with other population genetic parameters. Similarly, a mutation is pleiotropic if it affects trait expression at different organismal time points: fecundity early in life and age-related survivorship late in life, for example, a phenomenon that underlies the theory of antagonistic pleiotropy in life history evolution; or if it affects the expression of fitness-related traits in different environments. We know these phenomena are pervasive because of the preponderance of observations of genotype-by-sex interactions, genetic correlations among traits, and genotype-by-environment interactions.

### 2.4. Conditional Neutrality Harbors Cryptic Variation

One form of genotype-by-environment interaction is the phenomenon of conditional neutrality. Here, an allele segregating in a natural population will exhibit an effect on phenotype in one environment but no effect in another. This pattern has been commonly described in field studies investigating the genetic basis of local adaptation [50], and provides a crucial solution to intermixing populations for which local habitats vary in selection pressures. Because they can segregate neutrally in non-adaptive conditions, they are potentially still available when they arise in the right conditions. The alternative, if alleles are penetrant under all conditions, is ubiquitous exposure to natural selection, and alleles mismatched to their environment are more likely to be lost following negative selection. Further, accumulation of “cryptic” alleles—alleles that are silent under most conditions—may allow new mutations to rise to appreciable frequencies under drift, thus providing a store of natural genetic variation upon which selection may act in changing environments [2,51]. On the other hand, populations could theoretically (and do occasionally) solve the problem of variable environments by becoming plastic, in which the optimum phenotype is induced during the individual’s development rather than by the individual’s genotype. If this solution reduces standing variation in the population, it would necessarily limit future evolution, but the interplay between cryptic variation and plasticity is also potentially reinforcing [12].

Direct experimental evidence for the pervasive nature of condition-dependent genetic effects comes from response-eQTL studies. First described in nematodes, this is the observation that genetic polymorphisms that associate with gene expression may only have their effect in a certain environment [52]. Specifically, 59% of 308 trans-acting eQTL were temperature-specific, compared with 8% of 188 cis-acting eQTL. In the human immune system, we now know that more than half of all expressed genes are regulated by cis-acting eQTL, but the identities of the actual variants are different in lymphocytes, monocytes, dendritic cells, and neutrophils [53,54]. Importantly for our argument, they also vary after *ex vivo* perturbation by exposure to cytokines of immune stimulants such as lipopolysaccharide. It is now thought that the environment of a cell, which can include the microbiome, metabolic state, and exposure to pathogens or toxins, establishes a wide network of condition-specific regulation of gene activity. Similarly, the landscape of eQTL has been shown to differ between the normal colon and colorectal cancer. Here, an enrichment of novel mutations in the regulatory elements that contain binding sites for four different transcription factors which are themselves induced in the cancer cells, provides a direct mechanism for the condition dependence of genetic variation that only becomes relevant as disease progresses [55]. It is possible that the condition-dependent nature of gene expression variation observed here as polymorphic variation within a species potentiates evolution across taxa; the field of evo-devo has routinely emphasized the importance of regulatory changes between species, particularly those in cis [56].

Conditional neutrality can also occur as a function of an interaction between genotypes at two or more loci. By definition, this is statistical epistasis, measured as a departure from additive effects across loci in a population. Although epistasis probably does not contribute substantially to standing phenotypic variation [57], genetic interactions could be a primary mechanism by which cryptic variation may be exposed in individuals. For example, a novel allele may be penetrant in one genetic background, but masked in another, or admixture may generate novel combinations of alleles that generate previously unobserved phenotypes. The most important body of literature on this subject describes such scenarios in the context of “capacitor” genes, like the chaperone protein HSP90. Under this model, activity of the capacitor—e.g., chaperoning of polypeptide folding, localization of proteins and other homeostatic functions—runs interference between molecular variants and their reach to phenotype [58]. Disruption of capacitor function reveals their effects [59]. In the lab, subsequent selection on exposed phenotypes can lead to eventual fixation of the trait in the absence of the capacitor perturbation, an observation first reported by Waddington [60], and dubbed genetic assimilation. Gibson and Hogness [61] showed that artificial selection on cryptic variation in the key homeotic developmental regulatory gene *Ubx* contributed to genetic assimilation of the bithorax phenocopy, demonstrating how standard quantitative genetic processes can explain what to many was a very surprising phenomenon: That release of cryptic genetic variation can potentiate adaptive evolution. Recently, new work has challenged conventional ideas about cryptic variation [23]. In the case of the chromatin regulator HTZ1, new mutations are both masked and revealed by both the presence and absence of capacitor function, in equal degrees [62]. The allelic variants still exhibit (extensive) conditional neutrality, but the concept of a capacitor has been somewhat toppled; the cryptic alleles may find exposure under many scenarios.

### 2.5. Developmental Systems are Canalized

Canalization is evolved robustness [63]. The notion is that under persistent stabilizing selection, developmental or physiological systems evolve not just toward the optimal trait for the species, but also toward a genetic architecture that tends not to produce abnormal individuals. The major difficulties with accepting this idea are (i) that it theoretically requires selection on the epistatic component of genetic variation, which on the face of it is a violation of the fundamental conclusion that it is the additive component that responds to selection [64]; and (ii) that it involves study of the variance of variance, a notoriously difficult undertaking. Nevertheless, classical results, such as Dun and Fraser’s finding [65] that *Tabby* mutant mice do not just have fewer secondary vibrissae than wild-type ones (almost always 18 or 19 per side of the snout), but much higher variance (between 12 and 20), tell us that perturbation can affect not just the mean, but robustness about the mean. Unfortunately, there have been few follow-up studies of a similar nature. Gibson and van Helden [66,67] showed that the variance in haltere size in *Drosophila* increases in the presence of a *Ubx* mutation, and the variance for body weight in humans is greater in individuals with the high-BMI allele at the *FTO* locus [68]. Even though studies assessing variance in specific genotype-by-environment interactions generally yield negative results, there is an emerging literature on genetic risk score-by-environment effects suggesting that the summation of small interactions across many loci could yield substantial increases in variability for those at highest genetic risk who also have poor diets or behavioral patterns [69,70]. This is supported by simulation studies [71], and one of us has further proposed that decanalization, namely the increase in phenotypic variation that is brought about in novel environments or the presence of de novo mutations, may be a major contributor to rising incidence of chronic human disease [72].

Furthermore, we and others have consistently shown that there are vast pools of genetic variation affecting the cryptic phenotypes that are exposed by genetic or environmental perturbation. This includes homeotic and cancer-related phenotypes [73], embryogenesis [74], and developmental traits, such as body size [75] and pelvic girdle [76] in sticklebacks, vulva formation in worms [77], and eye development in cave fish [78]. Recently, there has been interest in variance eQTL, namely genotypes that influence variability in gene expression [79]. It is not yet clear whether such a molecular mechanism translates to phenotypic variance, but remarkably, Metzger *et al.* [80] showed that naturally occurring polymorphism in the promoter of the *TDH3* gene in yeast seems to have been more influenced by selection against expression noise than transcript abundance *per se*.

All of this is consistent with the notion that under persistent stabilizing selection, there is a tendency for genetic systems to become more robust. What is needed to prove that canalization is more than an obscure theoretical notion, are more field studies demonstrating that variance changes under different environmental or genetic circumstances. We suspect that systematic meta-analysis of thousands of published “common garden experiments” would in fact reveal substantial signals of condition-dependent variability. Even better would be comparisons of sister-species in different ecological niches, or of ancestor-progeny species pairs. For now, canalization, like Wright’s shifting balance theory [6], remains an intriguing evolutionary concept with considerable potential to help explain the micro-macro evolutionary nexus, but one whose role in evolution is exceedingly difficult to prove. We have accrued ample circumstantial evidence that genetic variation can be buffered, but it has not yet been established that this process potentiates adaptation.

### 2.6. Developmental System “Drift”

The shared evolutionary history of all living systems is an essential justification for the use of model organisms: Studying gene function in flies and mice can provide insight into their human orthologs, for example. However, conservation of sequence identity does not always indicate conservation of function. The phenomenon of developmental system drift occurs when developmental and morphological traits remain relatively static across evolutionary time, but the genetic underpinnings that encode them, including the functions and network interactions of conserved genes, diverge [81]. This is no better exemplified than in nematode worms, the phylum that includes *C. elegans*. The 25,000 known nematode species (and likely millions of existing species) exhibit a simple body plan and consistent wormlike appearance, but their genetic diversity places their last common ancestor as more ancient than that shared by humans and lampreys [82].

How can genes, genic functions and genetic interactions change so dramatically over evolutionary time while producing a relatively static developmental output? Experimental investigations into nematode biology have begun to characterize the different mechanisms by which similar traits are achieved. For example, vulva formation occurs by EGF/RAS signaling in *C. elegans* and WNT signaling in *P. pacificus*, nematode species that are 250 million years diverged. The molecular changes to these cell fate specifications include rewiring of the WNT pathway, acquisition of novel protein domains, and retention of conserved domains, indicating that both signal transduction lability and protein modularity can participate in developmental system drift [83]. At approximately 20 million years diverged, *C. elegans* and *C. briggsae* are near-identical in development and morphology, but orthologous genes show different molecular functions, often including differences in expression [84]. Although the term “developmental system drift” does not presuppose any particular mechanism, it has been hypothesized that stochasticity, including genetic drift, plays a dominant role in such evolutionary changes [81]. Divergence must begin with variation within populations, or at least with variation within the species. Despite extremely stereotyped embryonic development, wild-type *C. elegans* strains do show heritable differences in embryonic pathway function, as evidenced by differences in embryonic lethality following perturbation of critical developmental genes [74]. While embryogenesis appears robust under normal circumstances, these genetic differences in molecular function may represent latent opportunities for alternative developmental trajectories, paths that will be taken, or not, depending on the stochastic and deterministic forces to which populations are subject.

## 3. Conclusions

Natural genetic variation is the central focus of evolutionary and quantitative geneticists. However, within the paradigm of most investigations into developmental mechanisms, natural genetic variation becomes genetic background effects. Developmental geneticists often experience the perils of background effects, so virtually all forward- and reverse-genetic experiments are performed in common, isogenic strains. However, while this practice affords careful control of the focal perturbation, it also limits the reach of inference. Since the penetrance of single-gene mutations can vary dramatically across different genetic backgrounds, observations in a single strain may not be representative [85]. Moreover, the variability of a system is a critical feature of its function that can yield insight into its mechanisms [86], and mutations segregating in different genetic backgrounds can themselves be used to identify new genes for traits of interest [87,88]. Studying natural genetic variation complements laboratory-derived mutation approaches, by simultaneously assessing many variants within a systems-level perspective [89] and also by exploring perturbations that are mild to moderate, which may be required to be able to see their effects [90,91]. Moreover, non-model systems have always been important targets of study in molecular evolution, and the accessibility of genomic sequencing now nearly eliminates historical barriers to genetic studies of any organism.

The current accessibility of genotyping and sequencing technologies has turned the heads of many developmental biologists towards natural genetic variation, and we are extremely enthusiastic about the promise of such research programs. What we have argued here—that the robustness and lability of developmental systems arise from pervasive cryptic genetic variation, which may explain macro-evolutionary transitions and can be parsed at the micro-evolutionary scale by quantitative genetic methods—is simultaneously relevant to our understanding of mechanisms of development. The implications for understanding complex trait evolution are extensive, and include recognition that there is ample standing variation to suppress the deleterious effects of otherwise deleterious new mutations, that standing and cryptic variation should facilitate rapid response to environmental (including climate) change, and that highly buffered pathways allow tinkering of regulatory mechanisms that may contribute to the emergence of novelty. At the very least, rather than just treating alleles as statistical effects, quantitative genetics needs to be aware of the nature of developmental mechanisms, and molecular evolution needs to model the trajectory of genetic divergence.
